# Scalable Synthesis of Microsized, Nanocrystalline Zn_0.9_Fe_0.1_O‐C Secondary Particles and Their Use in Zn_0.9_Fe_0.1_O‐C/LiNi_0.5_Mn_1.5_O_4_ Lithium‐Ion Full Cells

**DOI:** 10.1002/cssc.202000559

**Published:** 2020-05-27

**Authors:** Jakob Asenbauer, Joachim R. Binder, Franziska Mueller, Matthias Kuenzel, Dorin Geiger, Ute Kaiser, Stefano Passerini, Dominic Bresser

**Affiliations:** ^1^ Helmholtz Institute Ulm (HIU) 89081 Ulm Germany; ^2^ Karlsruhe Institute of Technology (KIT) 76021 Karlsruhe Germany; ^3^ Institute for Applied Materials Karlsruhe Institute of Technology (KIT) 76344 Eggenstein-Leopoldshafen Germany; ^4^ Central Facility for Electron Microscopy Group of Electron Microscopy of Materials Science Ulm University Albert-Einstein-Allee 11 89081 Ulm Germany

**Keywords:** electrochemistry, conversion/alloying materials, lithium-ion batteries, particle design, spray-drying

## Abstract

Conversion/alloying materials (CAMs) are a potential alternative to graphite as Li‐ion anodes, especially for high‐power performance. The so far most investigated CAM is carbon‐coated Zn_0.9_Fe_0.1_O, which provides very high specific capacity of more than 900 mAh g^−1^ and good rate capability. Especially for the latter the optimal particle size is in the nanometer regime. However, this leads to limited electrode packing densities and safety issues in large‐scale handling and processing. Herein, a new synthesis route including three spray‐drying steps that results in the formation of microsized, spherical secondary particles is reported. The resulting particles with sizes of 10–15 μm are composed of carbon‐coated Zn_0.9_Fe_0.1_O nanocrystals with an average diameter of approximately 30–40 nm. The carbon coating ensures fast electron transport in the secondary particles and, thus, high rate capability of the resulting electrodes. Coupling partially prelithiated, carbon‐coated Zn_0.9_Fe_0.1_O anodes with LiNi_0.5_Mn_1.5_O_4_ cathodes results in cobalt‐free Li‐ion cells delivering a specific energy of up to 284 Wh kg^−1^ (at 1 C rate) and power of 1105 W kg^−1^ (at 3 C) with remarkable energy efficiency (>93 % at 1 C and 91.8 % at 3 C).

## Graphical abstract

**CAM anodes**: A new, scaled‐up synthetic route including three spray‐drying steps that results in the formation of microsized secondary particles of carbon‐coated Zn_0.9_Fe_0.1_O composed of nanocrystalline particles is described. This conversion/alloying material (CAM) provides very good electrochemical performance in Li‐ion half‐cells, and combination with LiNi_0.5_Mn_1.5_O_4_ (LNMO) cathodes results in Li‐ion cells showing remarkably high energy efficiency.

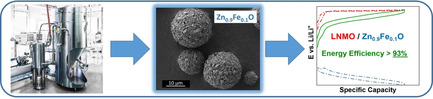

## Introduction

Since the first commercialization by Sony in 1991, the market for lithium‐ion batteries (LIBs) has been growing beyond expectations.^1^ This rapid increase in sales is essentially related to the superior energy and power density of LIBs, which have been and still are continuously improving thanks to the ongoing development of new materials or enhancement of existing materials and the steadily decreasing costs.^2^, ^3^, ^4^, ^5^, ^6^, ^7^ As a result, LIBs are nowadays employed not only in portable electronic devices, which was their initial target application, but also in stationary energy storage devices and pure and hybrid electric vehicles.^8^, ^9^, ^10^ This increasing diversity of potential applications, however, also leads to a greater variety of required characteristics. For example, the use in hybrid electric vehicles requires high power density while maintaining high safety and energy density as well as low cost.^11^ In addition, because of the rapid increase of production associated with the rapidly growing electrification of the automotive sector,^8^, ^12^ LIBs have to become more sustainable. This means that critical elements such as cobalt must be omitted, which concerns basically the cathode. Also, the whole production process must become environmentally friendly, for instance, by enabling the use of water as solvent for the preparation of both the negative (anode) and the positive (cathode) electrodes.^12^, ^13^, ^14^, ^15^ Accordingly, cobalt‐free cathode materials such as the high‐voltage spinel LiNi_0.5_Mn_1.5_O_4_ (LNMO), initially reported by Amine et al.^16^ and Zhong et al.,^17^ have attracted increasing interest. In fact, LNMO additionally provides excellent fast charging characteristics due to the 3D Li^+^ diffusion pathways in the spinel structure, while the high delithiation/lithiation potential of approximately 4.7 V ensures high energy and power densities.^18^ Nevertheless, the rate capability of the final full cell is commonly not determined by the cathode, but rather by the sluggish lithiation kinetics of the graphite anode.^19^ To improve the rate performance, several alternative anode materials have been developed and investigated, of which alloying‐type and conversion‐type compounds are the most promising with regard to energy density.^20^, ^21^, ^22^, ^23^, ^24^ Recently, a third class of compounds, namely, so‐called conversion/alloying materials (CAMs),^25^ has attracted increasing attention. CAMs combine both reaction mechanisms in a single material by in situ formation of nanograins of an alloying element and a percolating conductive network of transition metal nanograins on lithiation; the latter even allow for reversible cycling of the simultaneously formed Li_2_O matrix.^25^ One of the most investigated CAMs is Zn_0.9_Fe_0.1_O.^26^, ^27^, ^28^, ^29^, ^30^, ^31^ Besides being composed of environmentally friendly and abundant elements, it offers a high specific capacity of 966 mAh g^−1^ and very good rate capability. For both advantageous properties, however, the use of nanosized particles is essential, which is an obstacle for the realization of high‐density electrodes and, thus, suitable volumetric energy densities and their handling on an industrial scale, that is, their potential application in commercial devices.

Herein, we report a new, scalable synthesis route involving three spray‐drying steps that allows for the preparation of microsized but nanocrystalline carbon‐coated Zn_0.9_Fe_0.1_O (Zn_0.9_Fe_0.1_O‐C) secondary particles. The nanometric crystallite size of the primary particles ensures good electrochemical performance, while the large secondary particle size of about 10–15 μm facilitates handling and processing. The subsequent combination of this material as negative electrode with an LNMO‐based positive electrode enabled the first full cells of this kind showing potentially high energy efficiency and suitable specific energy.

## Results and Discussion

### Synthesis and characterization of carbon‐coated Zn_0.9_Fe_0.1_O

**Figure 1:**
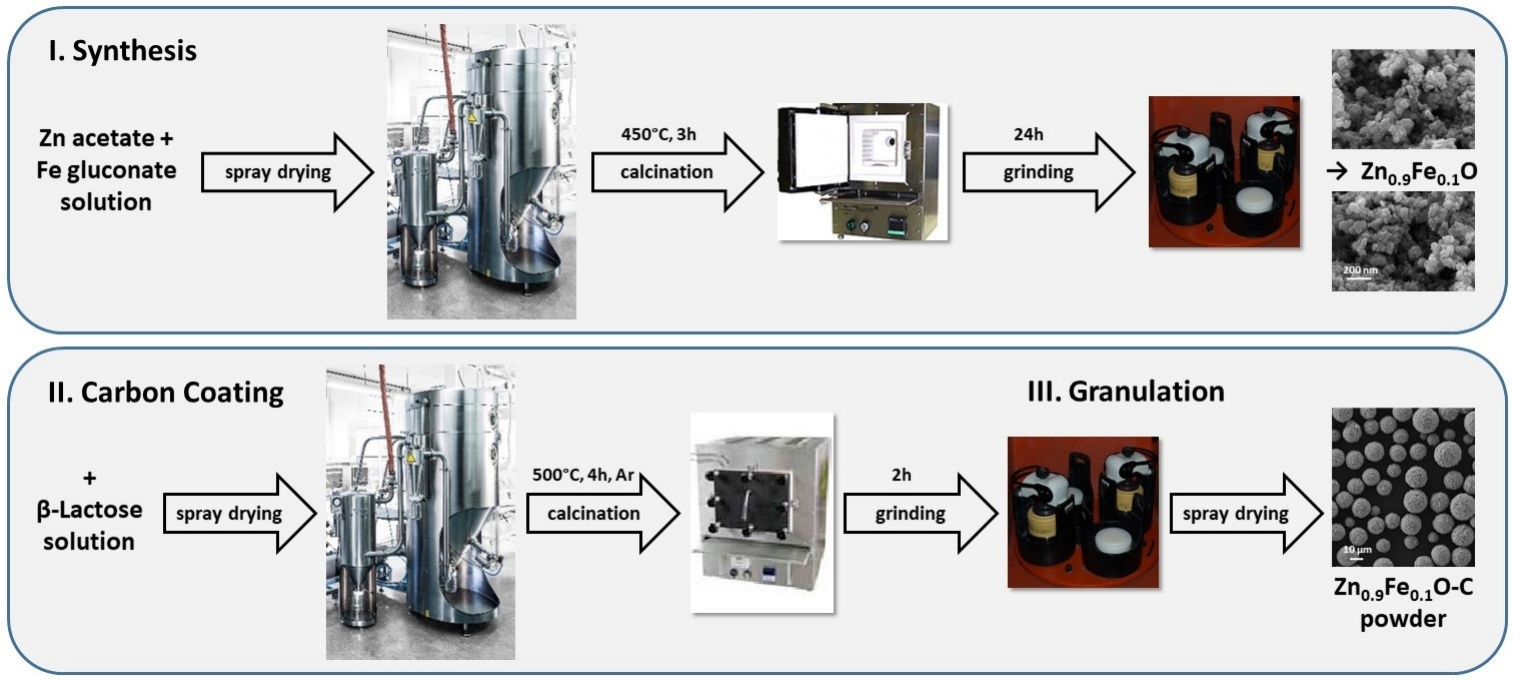
Representation of the scalable synthetic method for preparing Zn_0.9_Fe_0.1_O nanoparticles (I) and carbon‐coated, microsized Zn_0.9_Fe_0.1_O secondary particles (II).

**Figure 2:**
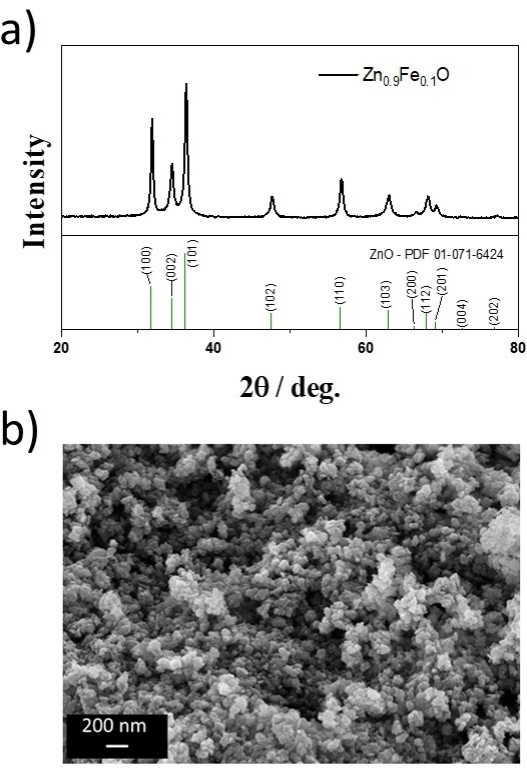
a) XRD pattern and b) SEM image of Zn_0.9_Fe_0.1_O nanoparticles, as obtained by step I of Figure 1 . The PDF reference for hexagonal wurtzite‐structured ZnO (PDF 01‐071‐6424) is provided at the bottom of a).

Carbon‐coated Zn_0.9_Fe_0.1_O (Zn_0.9_Fe_0.1_O‐C) was synthesized by a newly developed and readily scalable synthesis method, as summarized in Figure 1. Firstly, Zn_0.9_Fe_0.1_O nanoparticles were synthesized by spray drying of an aqueous solution of zinc(II) acetate and iron(II) gluconate precursors (9:1 molar ratio), both of which are rather cost‐efficient and environmentally friendly chemicals. The obtained compound was calcined in a box furnace at 450 °C for 3 h to give phase‐pure, wurtzite‐structured Zn_0.9_Fe_0.1_O nanoparticles (Figure 2 a). These have an average particle size of about 30 nm (Figure 2 b) and BET surface area of 33.5 m^2^ g^−1^. Compared with the laboratory‐scale synthesis reported earlier,^28^ this corresponds to a slight increase in particle size (formerly, <20 nm) and decrease in BET surface area (formerly, 90 m^2^ g^−1^). While these slight differences are certainly related to the different synthetic method, the choice of the precursors deserves brief reconsideration. In fact, the laboratory‐scale synthesis involving the gluconate salts of both metals^26^ leads to a significant volume expansion on synthesis due to the formation of a voluminous “foam” when the temperature is increased. For larger batches, this is rather hard to handle. Using the acetate salts instead can successfully address this issue, but results in the formation of impurity phases in the final product, so that it was not possible to reach homogeneous doping of Fe in the ZnO lattice (Figure S1 in the Supporting Information). In contrast, the combination of zinc acetate and iron gluconate allowed for the synthesis of a phase‐pure material while suppressing the extensive foaming. Similarly, the carbon coating procedure had to be adapted. In fact, the use of sucrose as carbon precursor turned out to be challenging. The wet grinding of the active material with sucrose leads to the formation of a soufflé‐like foam, and the rather pronounced hygroscopic nature of sucrose in combination with its relatively low glass transition temperature limit the potential processing window for the subsequent spray‐drying step.^32^ These issues could be tackled by replacing sucrose with β‐lactose and dispersing the active material without an additional grinding step in the ethanolic solution of β‐lactose, followed by spray drying the resulting dispersion. After calcination of the dried dispersion at 500 °C under argon atmosphere, the powder was ground and subsequently granulated in an additional spray‐drying step.

**Figure 3:**
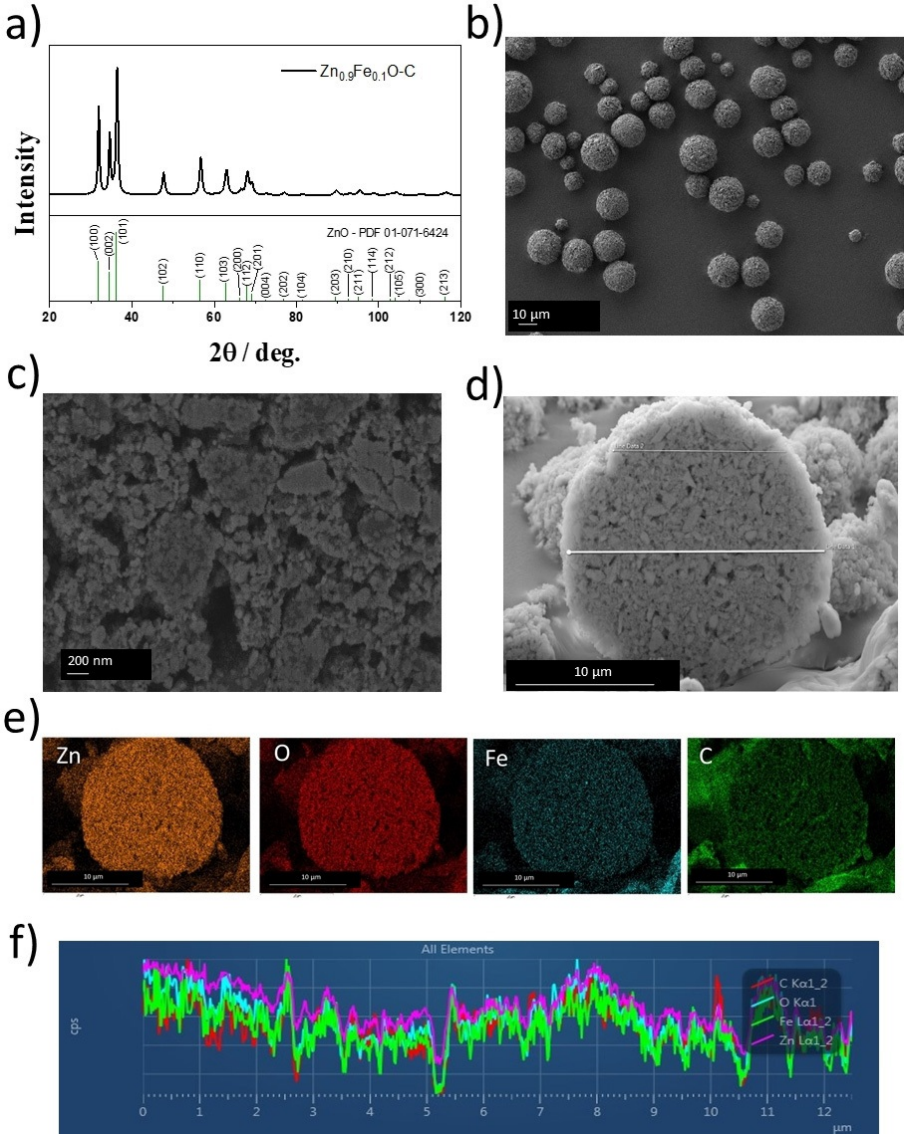
a) XRD pattern of the Zn_0.9_Fe_0.1_O‐C powder. The PDF reference for hexagonal wurtzite‐structured ZnO (PDF 01*‐*071*‐*6424) is provided at the bottom. b) SEM image of the finally obtained Zn_0.9_Fe_0.1_O‐C powder. c) SEM image of the cross section of a secondary particle at high magnification to illustrate the size and morphology of the primary nanoparticles. d) Cross section of a single secondary particle at lower magnification. e) EDX mapping for Zn (orange), oxygen (red), iron (blue), and carbon (green) and f) the normalized concentration of these elements along the horizontal white line in d).

**Figure 4:**
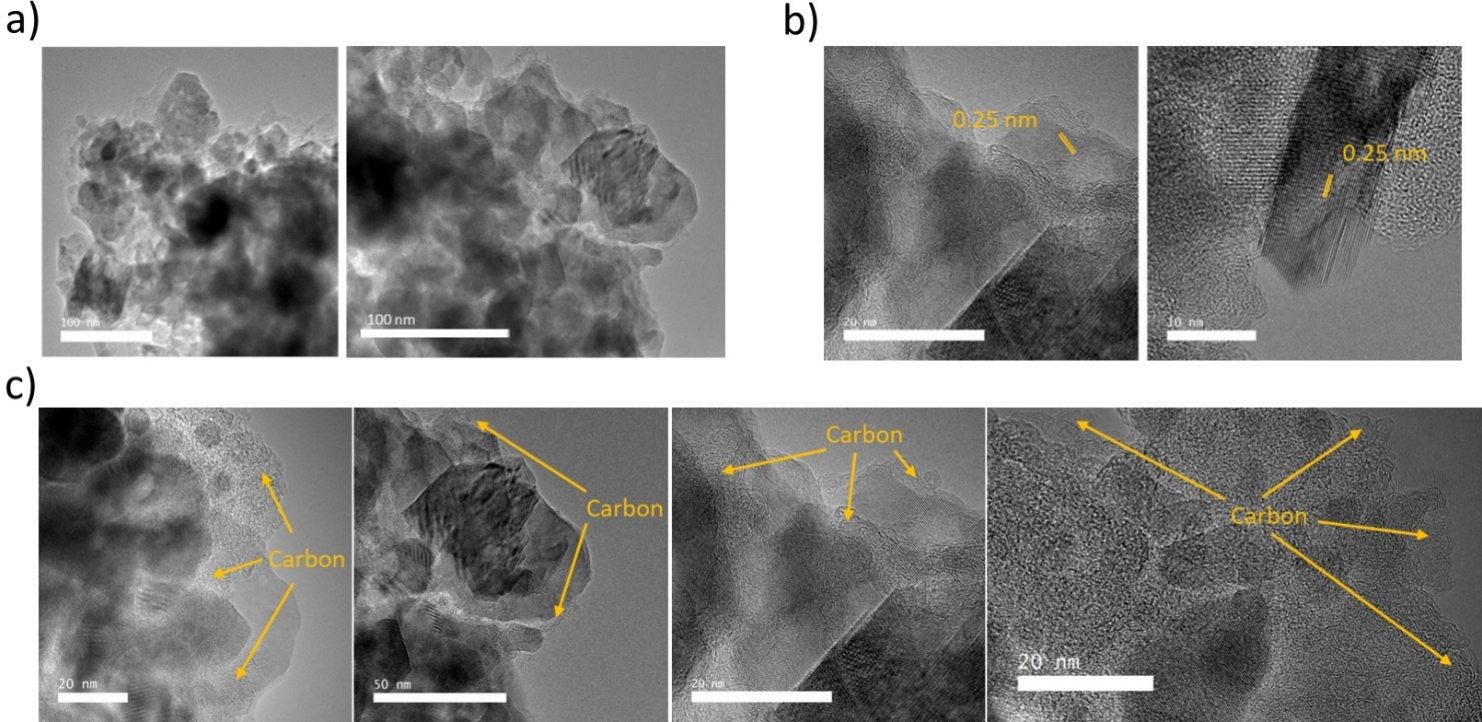
(HR)TEM analysis of the Zn_0.9_Fe_0.1_O‐C powder at different magnifications with a focus on a) the analysis of the particle size distribution of the primary particles, b) their crystallinity, and c) the distribution of the amorphous carbon coating within the secondary particles and at their surface.

The XRD pattern of the resulting Zn_0.9_Fe_0.1_O‐C is shown in Figure 3 a. No additional reflections are observed, and this confirmed that no phase impurities were introduced during the carbon‐coating process, while the width of the reflections is comparable to that of the uncoated Zn_0.9_Fe_0.1_O, that is, the particle size did not increase during the additional heat treatments. The total carbon coating content was determined by thermogravimetric analysis (TGA) to be approximately 13 wt % (Figure S2). The morphology of the granulated material was studied by SEM (Figure 3 b), which revealed that the Zn_0.9_Fe_0.1_O‐C powder consists of spherical secondary particles with a diameter of approximately 10–15 μm. These relatively large particles are composed of nanometric primary particles in the range of about 30–40 nm on average, as is apparent from the SEM image shown in Figure 3 c, which further confirms that the initial particles prior to carbon coating were well maintained. The cross section of a single granule is shown in Figure 3 d, which shows that these microsized secondary particles are composed of densely packed agglomerates of the nanocrystalline primary particles with a size of less than 1 μm. The pores between these agglomerates may facilitate electrolyte penetration into the secondary particles and, thus, favor the discharge/charge kinetics, though the determined true density of 4.1 g cm^−3^ is, as a consequence (in the case of inaccessible pores) and as a result of the carbon content (≈13 wt %), somewhat lower than the theoretical value for pure ZnO (5.6 g cm^−3^). The energy dispersive X‐ray (EDX) spectroscopic mapping for Zn, O, Fe, and C (Figure 3 e) as well as the longitudinal (normalized) elemental analysis (Figure 3 f) along the horizontal white line shown in Figure 3 d reveal that all elements are homogeneously distributed in these secondary particles. Considering carbon, this means that the single nanocrystals are also electronically well connected, and this suggests that ions and electrons can move rapidly from the outer shell into the core of the microsized secondary particles, which is essential for achieving high power. To obtain more detailed information about the morphology and structure of the carbon‐coated Zn_0.9_Fe_0.1_O material, we performed HRTEM (Figure 4). Figure 4 a shows two micrographs at the outer edge of a secondary particle for studying the size of the primary nanocrystals. Globally, a size distribution of about 15–80 nm is observed, with the majority of the particles having a diameter of approximately 30–40 nm, in line with the SEM observation. In Figure 4 b two additional micrographs at higher resolution reveal that the primary particles are highly crystalline with fringes of, for example, about 0.25 nm for the interlayer spacing of the (101) planes^33^ (highlighted in yellow). For the HRTEM image in Figure 4 c, the focus was on studying the distribution of the carbon coating at the local scale. It is apparent that the amorphous carbon (in line with the absence of any additional carbon‐related reflection in Figure 3 a) thoroughly interconnects the single primary particles at the corresponding interfaces and largely covers the surface of the primary particles with a layer of several nanometers (exemplarily illustrated by the yellow arrows). It is also observed, however, that some primary particles are not fully covered by carbon at the outer surface of the secondary particle. Nonetheless, this does not hamper the electron transport in the secondary particles.

### Electrochemical characterization

**Figure 5:**
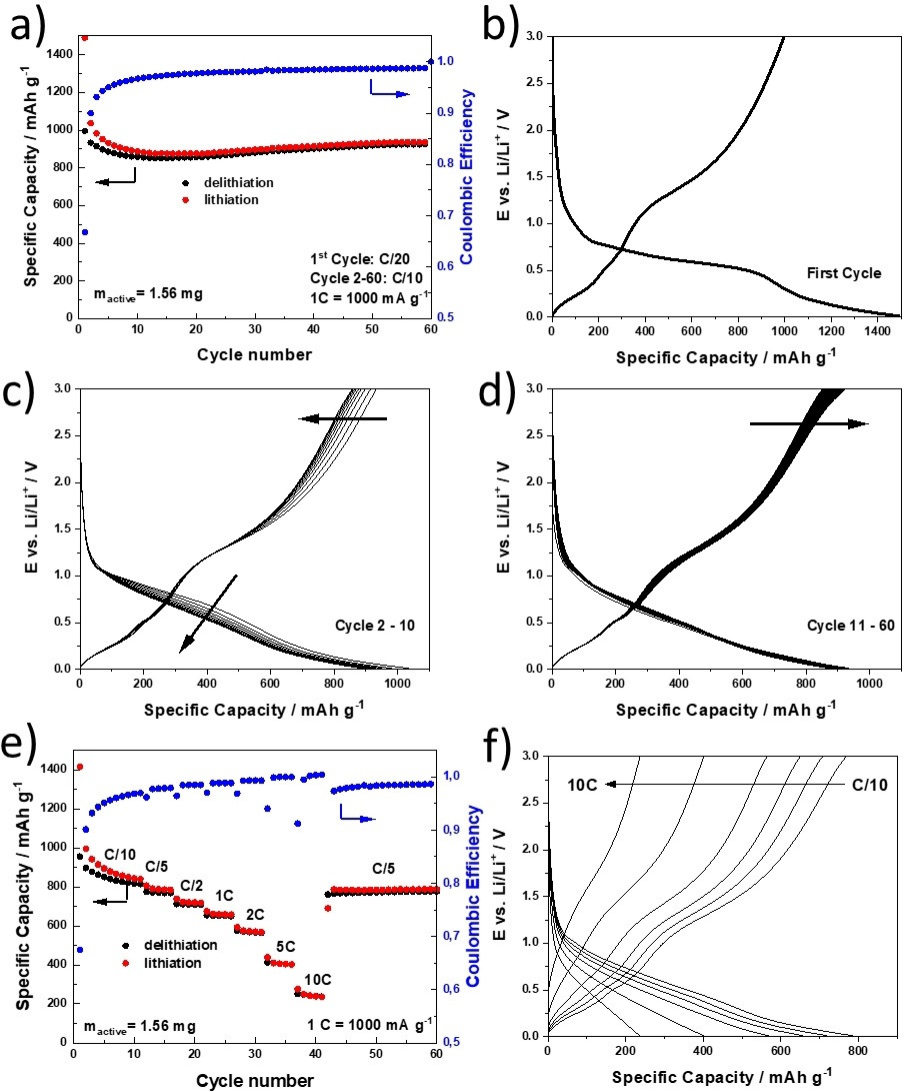
a) Plot of capacity versus cycle number for Zn_0.9_Fe_0.1_O‐C/Li half‐cells subjected to a specific current of 100 mA g^−1^ after the first formation cycle at 50 mA g^−1^ (cutoff voltages: 0.01 and 3.0 V). b–d) The corresponding discharge/charge profiles for b) the first cycle, c) cycles 2–10, and d) cycles 11–60. e) Plot of capacity versus cycle number for Zn_0.9_Fe_0.1_O‐C/Li half‐cells subjected to elevated discharge/charge rates ranging from C/10 to 10C (i.e., specific currents of 100 to 10 000 mA g^−1^; cut‐off voltages: 0.01 and 3.0 V) and f) the corresponding discharge/charge profiles. All specific capacities refer to the mass of the active material including the carbonaceous coating.

For the electrochemical characterization of Zn_0.9_Fe_0.1_O‐C, half‐cells assembled with lithium metal as counter electrode were subjected to galvanostatic cycling (Figure 5). For this basic characterization, the whole voltage range of the electrochemical activity of Zn_0.9_Fe_0.1_O was explored. Firstly, we evaluated the constant‐current cycling at a relatively low specific current (100 mA g^−1^) after one formation cycle at 50 mA g^−1^ (Figure 5 a). The coulombic efficiency in the first cycle is approximately 70 % (see also Figure 5 b) but increases to about 97–99 % later, depending on the C rate (Figure 5 a). The reversible specific capacity decreases in the initial ten cycles (Figure 5 a and c). However, on further cycling it stabilizes at about 850 mAh g^−1^ and even tends to slightly increase later on (Figure 5 a and d). Such a trend, observed earlier for other conversion and conversion/alloying materials, has been assigned to the quasireversible formation of the solid electrolyte interphase (SEI) layer.^34^ This is also supported by the capacity increase essentially occurring at rather high and low potentials on charge and discharge, respectively.^35^, ^36^, ^37^, ^38^, ^39^ We note, however, that this increase is only marginal compared to earlier studies,^36^, ^37^, ^38^ and this suggests that the carbon coating better stabilizes the active material/electrolyte interface, that is, suppresses the dissolution and reformation of the SEI. This quasireversible SEI formation can be effectively inhibited by limiting the upper cutoff voltage to 2.0 V or less, as confirmed by a recent study involving in situ microcalorimetry.^40^

The rate capability of the Zn_0.9_Fe_0.1_O‐C electrode was investigated by subjecting the cells to discharge/charge rates ranging from C/10 to 10C (i.e., specific currents ranging from 100 to 10 000 mA g^−1^; Figure 5 e and f). Before increasing the discharge/charge rate, the cells were cycled for ten cycles at C/10, that is, until the coulombic efficiency had stabilized at approximately 97 %. As the C rate increases from 100 to 200, 500, 1000, 2000, 5000, and 10 000 mA g^−1^, the capacity decreases from 810 to 770, 710, 650, 410, and 240 mAh g^−1^, respectively, indicating very good rate capability of the microsized, nanocrystalline Zn_0.9_Fe_0.1_O‐C. This is also reflected by the relatively small increase in polarization considering the currents applied (Figure 5 f). After this C‐rate test, the specific current was decreased again to 200 mA g^−1^ (C/5), which resulted in a capacity of approximately 770 mAh g^−1^, that is, the same value as before the C‐rate test at this specific current. This confirms the good reversibility of the delithiation/lithiation mechanism of Zn_0.9_Fe_0.1_O‐C.

Generally, these results clearly exceed the rate capability data reported earlier for the materials derived from laboratory‐scale synthesis.^26^, ^28^ For instance, some of us have previously reported specific capacities of approximately 450, 300, and 110 mAh g^−1^ at specific currents of 1000, 2000, and 5000 mA g^−1^, respectively,^28^ which are substantially lower than the capacities presented herein, that is, ca. 650, 410, and 240 mAh g^−1^, respectively. This brief comparison further highlights that the newly developed, scaled‐up synthesis does not have any negative, but instead a highly advantageous impact on the material performance.

### Zn_0.9_Fe_0.1_O‐C/LiNi_0.5_Mn_1.5_O_4_ lithium‐ion cells

**Figure 6:**
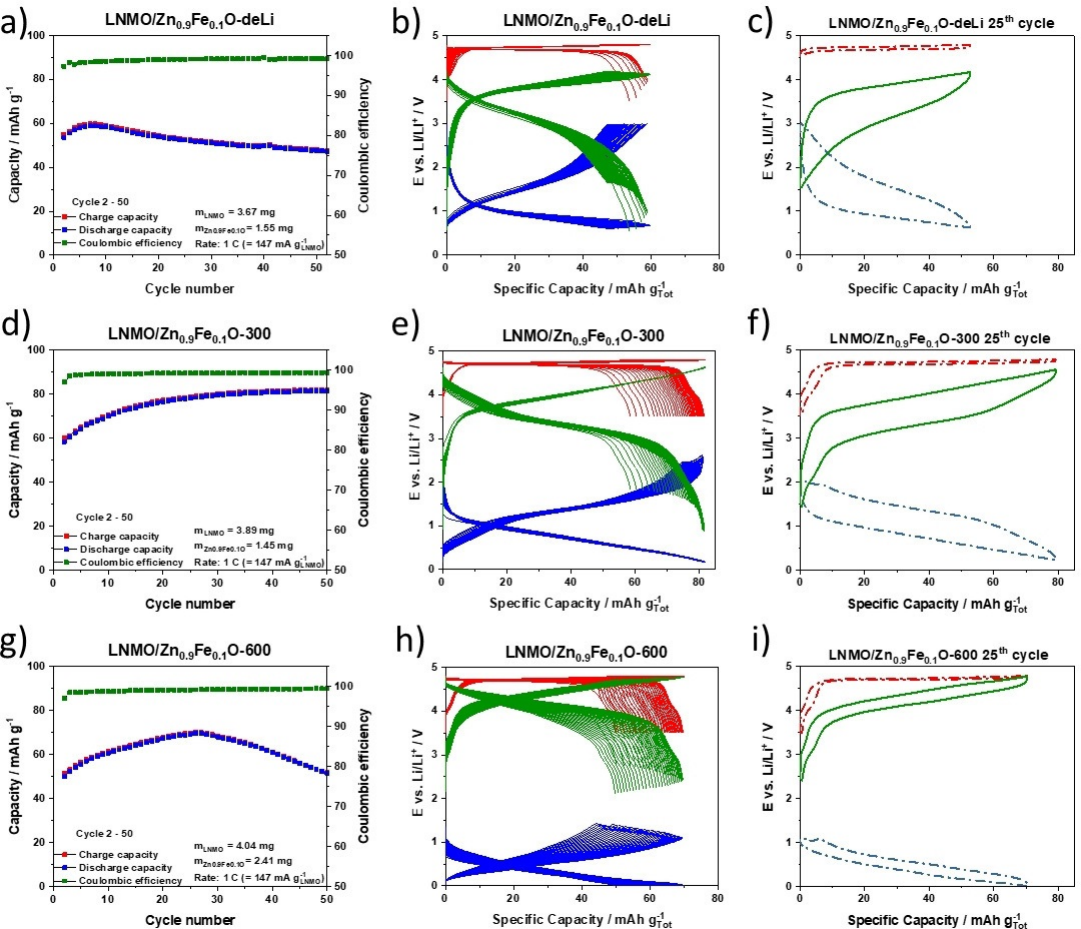
Galvanostatic cycling of Zn_0.9_Fe_0.1_O/LNMO full cells at 1 C (147 mA gLNMO-1 ) having anodes with different degrees of prelithiation. a–c) Zn_0.9_Fe_0.1_O‐deLi, d–f) Zn_0.9_Fe_0.1_O‐300, and g–i) Zn_0.9_Fe_0.1_O‐600. For each full cell the following plots are shown (from left to the right): plot of the specific capacity versus cycle number, the corresponding separate discharge/charge profiles for all cycles for the full cell (green), the anode (blue), and the cathode (red), and the slightly modified plot of the discharge/charge profile for the 25th cycle, highlighting the voltage hysteresis between the charge and discharge processes. Note that the specific capacities are based on the sum of the anode and cathode active materials. Prior to cycling at 1 C, a formation cycle at C/10 was applied to each cell. The potentials of the LNMO cathodes and the Zn_0.9_Fe_0.1_O anode were limited to 3.5–4.8 and 0.01–3.0 V, respectively.

To demonstrate the potential of Zn_0.9_Fe_0.1_O‐C as alternative anode material for high‐power Li‐ion batteries, we combined Zn_0.9_Fe_0.1_O‐C negative electrodes with high‐voltage LNMO positive electrodes. To the best of our knowledge, this is the first Li‐ion cell of such a kind. As the first cycle coulombic efficiency of Zn_0.9_Fe_0.1_O‐C still deserves further improvement, the negative electrodes were first prelithiated. To fine‐tune the operational potential range of the anode three differently prelithiated sets of electrodes were used, following a previous study.^41^ For the first one, the anodes were lithiated and completely delithiated prior to full‐cell assembly (Zn_0.9_Fe_0.1_O‐deLi; Figure 6 a–c). For the second one, the anodes were partially lithiated with a specific capacity of 300 mAh g^−1^ (Zn_0.9_Fe_0.1_O‐300; Figure 6 d–f), and for the third one the anodes were partially lithiated with a specific capacity of 600 mAh g^−1^ (Zn_0.9_Fe_0.1_O‐600; Figure 6 g–i). Note that the specific capacities given in Figure 6 refer to the mass of both active materials, that is, the negative and positive electrodes. For easier comparison of the performance of the cathode and anode individually, the same plot is provided in Figures S3 and S4, in which the specific capacities refer to the active‐material mass loading of the LNMO cathode and the Zn_0.9_Fe_0.1_O anode, respectively. For a graphite/LNMO cell with a N/P capacity ratio of 1.2, the theoretical specific capacity of the full cell would correspond to 80 mAh g^−1^ (assuming a reversible specific capacity of 350 mAh g^−1^ for the graphite anode). In the Li‐ion cells investigated herein, the P/N mass ratio was 2.36, 2.68, and 1.68, respectively for Zn_0.9_Fe_0.1_O‐deLi, Zn_0.9_Fe_0.1_O‐300, and Zn_0.9_Fe_0.1_O‐600. Considering the substantially higher capacity of the anode, this means that all cells were cathode‐limited with N/P capacity ratios of 3.42, 1.95, and 1.42 for the full cells with Zn_0.9_Fe_0.1_O‐deLi, Zn_0.9_Fe_0.1_O‐300, and Zn_0.9_Fe_0.1_O‐600 anodes, respectively. These values were calculated on the basis of the practically obtained capacities of these electrodes at the given discharge/charge rate, that is, 105 mA g^−1^ for LNMO and 850 mAh g^−1^, 550 mAh g^−1^, and 250 mAh g^−1^, for Zn_0.9_Fe_0.1_O‐deLi, Zn_0.9_Fe_0.1_O‐300, and Zn_0.9_Fe_0.1_O‐600, respectively. When cycled at 1 C (147 mA g^−1^), all cells showed an initial increase in capacity over several cycles (Figure 6 a, d, and g), which is related to a continuous increase in capacity for the LNMO cathode, as is apparent from the discharge/charge profile evolution. The steady increase in capacity occurs along the high‐voltage plateau (Figure 6 b, e, h) originating from the Ni^2+^⇄Ni^3+^⇄Ni^4+^ redox reaction.^32^ This already‐observed phenomenon has been assigned to the sluggish electrolyte wetting of the aqueous‐processed LNMO cathode.^42^

The full cell with Zn_0.9_Fe_0.1_O‐deLi as negative electrode (Figure 6 a) shows an initial increase in capacity, followed by slight but steady fading. The anode reaches immediately the upper cutoff voltage of 3.0 V, while the lower cutoff of the cathode rapidly rises, so that eventually only the nickel redox couple at about 4.7 V is utilized (Figure 6 b). In combination with the rather low coulombic efficiency (≈99 %) during the initial 15 cycles, this behavior indicates ongoing lithium loss. The discharge/charge profile for the 25th cycle (Figure 6 c) reveals that the anode does not reach its lower cutoff voltage and delithiation/lithiation occurs essentially in the regime of the conversion reaction.^26^, ^31^, ^40^ This results in a rather low energy efficiency (EE) of approximately 78.2 %, an average full‐cell voltage of 3.0 V, and a specific energy of 157 Wh kg^−1^ for the 25th cycle. Although such an EE is rather low compared to lithium‐ion cells with a graphite anode, it is significantly higher than that of only approximately 62 % reported earlier for (theoretical) ZnFe_2_O_4_/LiFePO_4_ full cells.^43^

To avoid the energy loss associated with the anode operating at high voltages, the prelithiated Zn_0.9_Fe_0.1_O‐300 electrode (300 mAh g^−1^) was employed in the full cell. This “lithium reservoir” allows for substantially higher capacities, which stabilize at approximately 80 mAh g^−1^ (i.e., the same specific full‐cell capacity as for a graphite/LNMO lithium‐ion cell), and cycling stability (Figure 6 d). The capacity‐limiting LNMO cathode cycles stably within the set cutoff potentials, so that the characteristic feature of the Mn^3+/4+^ redox reaction at approximately 4.1 V is well maintained (Figure 6 e). In fact, recalculating the specific capacity for the LNMO cathode gave a value of approximately 110 mAh gLNMO-1 , that is, the full capacity of the cathode is used. Similarly, the operational voltage range of the anode is substantially extended to lower potentials compared with the full cell based on Zn_0.9_Fe_0.1_O‐deLi, without hitting the upper cutoff potential of 3.0 V. Nevertheless, a slight increase is observed on cycling with a very particular feature occurring after about ten cycles. Towards the end of the discharge step, a voltage “bump” is recorded, which gets more pronounced on further cycling. Interestingly, its overlapping with the Mn^3+/4+^‐related voltage plateau of the cathode suggests that it is caused by the reduction and reoxidation of Mn^2+^ cations, formed at the positive electrode from the dismutation of Mn^3+^ into Mn^4+^ and soluble Mn^2+^, at the anode.^44^, ^45^, ^46^ This feature, which is considered to have a rather negative impact on the long‐term cycling performance and stability of the SEI at the anode,^47^, ^48^, ^49^ has not been observed for the Zn_0.9_Fe_0.1_O‐deLi/LNMO full cell, since the manganese redox process at the cathode did not occur extensively owing to the fast capacity fading. Accordingly, the LNMO cathode requires further improvement for operation in lithium‐ion cells. Nonetheless, the partial prelithiation allows for a significant increase of the average cell voltage (3.3 V), specific energy (262 Wh kg^−1^), and EE (83.2 %), as exemplarily determined for the 25th cycle at 1 C (Figure 6 f). In fact, the shift of the operational potential of the anode to lower values, accompanied by an increased share of the alloying contribution, results in an appreciable decrease of the voltage hysteresis and, thus, a higher EE.

**Figure 7:**
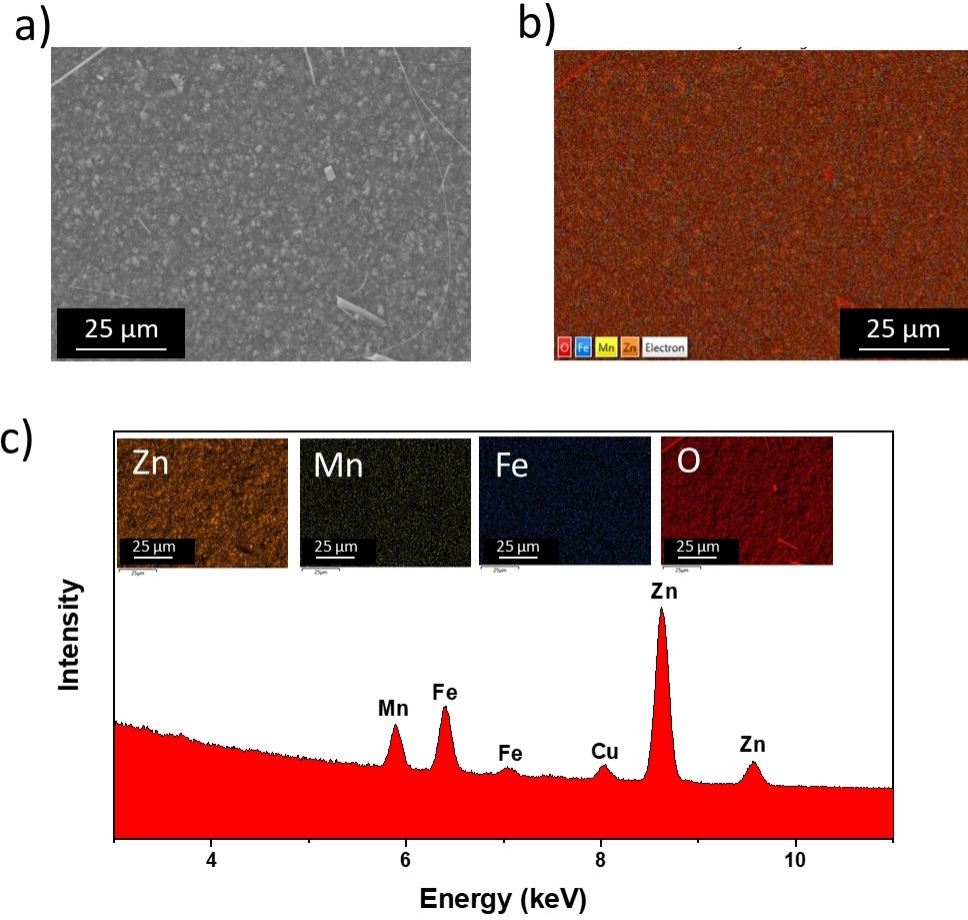
SEM/EDX analysis of the Zn_0.9_Fe_0.1_O‐600 negative electrode after cycling in Zn_0.9_Fe_0.1_O‐600/LNMO full cells (Figure 6 g–i). a) The corresponding SEM image. b) EDX mapping for Zn, Mn, Fe, and O together; c) EDX mapping for each element separately and the plot of the different elemental intensities versus their energies.

To explore the effect of more extensive prelithiation, we increased the lithium reservoir in the anode to 600 mAh g^−1^ (i.e., Zn_0.9_Fe_0.1_O‐600). Just as in the previous case, the capacity initially rises to approximately 70 mAh g^−1^ after 25 cycles (Figure 6 g). Although this corresponds again to a specific capacity of approximately 110 mAh g^−1^ for the cathode, that is, its complete utilization, the overall value is lower due to the larger anode and its lower (remaining) capacity after the prelithiation. Also, the overall cell capacity fades rather rapidly on cycling, even faster than that of the Zn_0.9_Fe_0.1_O‐deLi/LNMO full cell (Figure 6 a). Considering the large lithium reservoir, it appears unlikely that this fading is related to lithium loss. In fact, the voltage profile of the cathode is well maintained in its shape (Figure 6 h). Instead, the shape of the anode discharge/charge profile changes to a greater extent. In particular, the feature assigned to manganese reduction and reoxidation at the anode is more pronounced in this case, and this highlights its detrimental effect on the full‐cell performance. Indeed, the manganese concentration on the Zn_0.9_Fe_0.1_O‐600 anode is rather high, as confirmed by ex situ SEM/EDX analysis (Figure 7). Nonetheless, for the exemplary 25th cycle, the Zn_0.9_Fe_0.1_O‐600/LNMO lithium‐ion cell provides remarkable average voltage (4.1 V), specific energy (284 Wh kg^−1^, i.e., almost double the specific energy of the Zn_0.9_Fe_0.1_O‐deLi/LNMO cell), specific power (375 W kg^−1^), and EE (>93 %; Figure 6 i), a value that is comparable to those of state of the art graphite‐based Li‐ion cells.^43^ Since these values again refer only to the two active materials at the negative and positive electrodes, so that for direct comparison with commercial cells also the inactive components would have to be considered (commonly, extensive optimization is done prior to any commercialization). Nevertheless, the EE is not affected by the presence and any optimization of the inactive components (apart from polarization effects) and the specific energy reported herein is rather comparable to that of a little more than 300 Wh kg^−1^ recently reported for a graphite/LNMO laboratory‐scale full cell, albeit at a lower discharge/charge rate of C/3.^50^ A summary of the results obtained for the three full cells is provided in Table 1.

**Table 1:** Mass ratio, N/P ratio, specific energy, average discharge voltage, and energy efficiency of Zn_0.9_Fe_0.1_O/LNMO full cells with different degrees of prelithiation obtained from galvanostatic cycling at 1 C (147 mA $g_{\mathrm{LNMO}}^{-1}$ ).

Full cell	LNMO/Zn_0.9_Fe_0.1_O mass ratio	N/P	Specific energy [Wh kg^−1^]	Vavgdischarge [V]	EE [%]
Zn_0.9_Fe_0.1_O‐deLi/LNMO	2.36	3.42	157	3.0	78.2
Zn_0.9_Fe_0.1_O‐300/LNMO	2.68	1.95	262	3.3	83.2
Zn_0.9_Fe_0.1_O‐600/LNMO	1.68	1. 42	284	4.1	93.2

**Figure 8:**
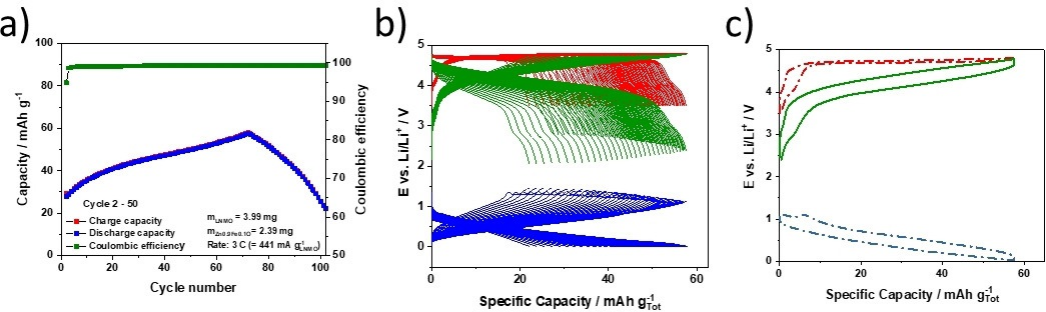
Galvanostatic cycling of Zn_0.9_Fe_0.1_O‐600/LNMO full cells at 3 C (441 mA gLNMO-1 ). a) Plot of the specific capacity versus cycle number. b) The corresponding discharge/charge profiles for all cycles for the full cell (green) and separate electrodes (red and blue for the cathode and anode, respectively). c) Slightly modified plot of the discharge/charge profile for the 70th cycle. Note that the specific capacities are based on the sum of those of the anode and cathode active materials. Prior to cycling at 3 C a formation cycle at C/10 was applied to the cell. The potentials of the LNMO cathodes and the Zn_0.9_Fe_0.1_O anode were limited to 3.5–4.8 and 0.01–3.0 V, respectively.

A high EE is, in fact, not only important with regard to general aspects such as sustainability and cost, but especially relevant for high‐power batteries, since energy inefficiency is largely released as heat,^40^ which might be an issue in practical application. Hence, we also subjected LNMO/Zn_0.9_Fe_0.1_O‐600 full cells to elevated discharge/charge rate (3 C) to study the impact of such increased current on the EE, while simultaneously evaluating the general applicability of such lithium‐ion cells for high‐power devices (Figure 8). Generally, the cell shows a similar behavior to that cycled at 1 C (Figure 6 g), that is, an initial increase up to about 58 mAh g^−1^ at the 70th cycle (corresponding to 92 mAh g^−1^ for the LNMO cathode only) and subsequent rapid decrease. While these results further highlight the need for an optimized cathode to suppress dissolution of manganese and its subsequent redox activity at the anode, the specific energy (230 Wh kg^−1^) and power (1105 W kg^−1^), EE (91.8 %), and average discharge voltage (4.0 V) make this lithium‐ion cell chemistry very suitable for high‐power applications.

## Conclusions

We have described a new, scaled‐up synthesis route for the preparation of carbon‐coated Zn_0.9_Fe_0.1_O composed of nanocrystalline particles agglomerated into microsized secondary particles. The material provides very good electrochemical performance in half‐cells with a reversible capacity of approximately 850 mAh g^−1^ at C/10 and high rate capability. The investigation of different degrees of prelithiation in Zn_0.9_Fe_0.1_O/LNMO full cells revealed the beneficial effect of limiting the operational potential of the anode to the alloying‐dominated regime while granting a lithium reservoir. As a result, these cells showed specific energies of up to 284 Wh kg^−1^ at 1 C and 230 Wh kg^−1^ at 3 C, corresponding to specific powers of 375 and 1105 W kg^−1^, respectively. Remarkably, the energy efficiencies at such discharge/charge rates are as high as >93 % (1 C) and 91.8 % (3 C), which suggest that this cell chemistry is generally suitable for high‐power applications if the manganese dissolution from the cathode can be suppressed.

## Experimental Section

### Material synthesis

To obtain microsized nanocrystalline Zn_0.9_Fe_0.1_O‐C, a two‐step synthesis was used. First, the zinc(II) acetate dihydrate (Alfa Aesar) and iron(II) d‐gluconate dihydrate (Aldrich) precursors were dissolved in water and the solution was spray dried with a GEA Niro Mobile Minor spray dryer to synthesize Zn_0.9_Fe_0.1_O nanoparticles. Subsequently, the powder was calcined at 450 °C for 3 h (VMK‐1400, Linn High Therm) and afterwards ground by planetary ball milling (Pulverisette 5, Fritsch) for 24 h by using yttria‐stabilized zirconia beads. The carbon coating was achieved by spray drying a dispersion of the Zn_0.9_Fe_0.1_O nanoparticles in an aqueous solution of β‐lactose, followed by calcination of the resulting powder at 500 °C for 4 h under an argon atmosphere (VMK‐135‐S, Linn High Therm). Finally, the carbon‐coated Zn_0.9_Fe_0.1_O nanoparticles (Zn_0.9_Fe_0.1_O‐C) were ground again by planetary ball milling for 2 h and granulated by spray drying.

### Structural and Morphological Characterization

The powder properties of the synthesized Zn_0.9_Fe_0.1_O were investigated by powder XRD (D5005, Siemens), field‐emission SEM (Supra 55, Zeiss), and nitrogen physisorption (Gemini VII 2390, Micromeritics). The XRD measurements were performed with Cu_Kα_ radiation in a 2*θ* range of 15–80°, and SEM images were obtained at an accelerating voltage of 10 kV. The specific surface area was calculated according to the BET theory. The structure of Zn_0.9_Fe_0.1_O‐C was studied by XRD with a Bruker D8 Advance diffractometer (Cu_Kα_ radiation, *λ*=0.154 nm) in the 2*θ* range of 20–90°. SEM was conducted with a Zeiss Crossbeam 340 field‐emission electron microscope, equipped with an EDX spectrometer (Oxford Instruments X‐MaxN, 50 mm^2^, 15 kV) and a Capella gallium‐focused ion beam (FIB). For the ex situ EDX measurements, the cycled electrodes were recovered in an argon‐filled glove box, carefully rinsed with dimethyl carbonate (DMC), and transferred to the SEM under argon atmosphere with a specially designed transfer box (Sample Transfer Shuttle, SEMILAB). For the FIB treatment, currents of 1.5 nA and 50 pA at an acceleration voltage of 30 kV were chosen for milling and polishing, respectively. HRTEM images where recorded with a Cs‐corrected high‐resolution transmission electron microscope (FEI Titan, 80–300 kV) operated at acceleration voltages of 80 and 300 kV. The weight of the carbon coating was determined by TGA (Model Q5000, TA Instruments) in the temperature range of 40–850 °C under an oxygen atmosphere. The true density of the carbon‐coated sample (Zn_0.9_Fe_0.1_O‐C) was determined by utilizing an AccuPyc II 1340 gas pycnometer and helium as working gas.

### Electrode preparation

For electrode preparation, Zn_0.9_Fe_0.1_O‐C and carbon black (Super C65, Imerys) were added to a 1.25 wt % solution of sodium carboxymethyl cellulose (CMC, Dow Wolff Cellulosics) in deionized water. The composition of the dry materials in the slurry was 75 wt % Zn_0.9_Fe_0.1_O‐C, 20 wt % carbon black, and 5 wt % CMC. The slurry was mixed by planetary ball milling (Pulverisette 4, Fritsch) for 2 h. The homogenized slurry was then cast on dendritic copper foil (Schlenk) by using a laboratory doctor blade with a wet‐film thickness between 120 and 200 μm and subsequently dried at 80 °C for 5 min and 12 h at room temperature. Disk electrodes (12 mm diameter) where punched and dried for 12 h at 120 °C under vacuum. The LNMO cathodes for full‐cell assembly where prepared as reported by Kuenzel et al.^42^

### Electrochemical characterization

The electrochemical characterization was performed in three‐electrode Swagelok‐type cells, assembled in an argon‐filled glove box (MBraun, Germany; oxygen and water content <0.1 ppm). As separator, a sheet of glass fiber fleece (Whatman, GFD), soaked with a 1 m solution of LiPF_6_ in a mixture of ethylene carbonate (EC) and diethyl carbonate (for the half‐cell tests) or EC and DMC (for the full‐cell tests with the LNMO cathodes) was used. In the half‐cells battery‐grade Li metal (Honjo) served as both counter and reference electrodes. The specific capacities provided herein are based on the mass of the active material including the carbon coating. For the full‐cell tests employing lithium metal as quasireference electrode, the Zn_0.9_Fe_0.1_O‐C anodes were galvanostatically precycled for ten cycles at C/10 (i.e., 0.1 A g^−1^) in the potential range of 0.01–3.0 V vs. Li/Li^+^. Eventually, the electrodes were partially lithiated, as indicated in the text. Subsequently, the cells were disassembled under argon, and full cells were assembled from such anodes and fresh electrolyte and separator.

## Conflict of interest

*The authors declare no conflict of interest*.

## Supporting information

As a service to our authors and readers, this journal provides supporting information supplied by the authors. Such materials are peer reviewed and may be re‐organized for online delivery, but are not copy‐edited or typeset. Technical support issues arising from supporting information (other than missing files) should be addressed to the authors.

Supplementary
