# Supplementary material for: Scalable Synthesis of Microsized, Nanocrystalline Zn0.9Fe0.1O‐C Secondary Particles and Their Use in Zn0.9Fe0.1O‐C/LiNi0.5Mn1.5O4 Lithium‐Ion Full Cells
Source: ChemSusChem. 2020 May 27;13(13):3504–13. doi: 10.1002/cssc.202000559 (PMC7384102; doi:10.1002/cssc.202000559)
Supplement: Supplementary file 1 — Supplementary [file CSSC-13-3504-s001.pdf]

ChemSusChem

Supporting Information

Scalable Synthesis of Microsized, Nanocrystalline $Zn_{0.9}Fe_{0.1}O$-C Secondary Particles and Their Use in $Zn_{0.9}Fe_{0.1}O$-C/$LiNi_{0.5}Mn_{1.5}O_4$ Lithium-Ion Full Cells

Jakob Asenbauer,[a, b] Joachim R. Binder,[c] Franziska Mueller,[a, b] Matthias Kuenzel,[a, b] Dorin Geiger,[d] Ute Kaiser,[d] Stefano Passerini,*[a, b] and Dominic Bresser*[a, b]

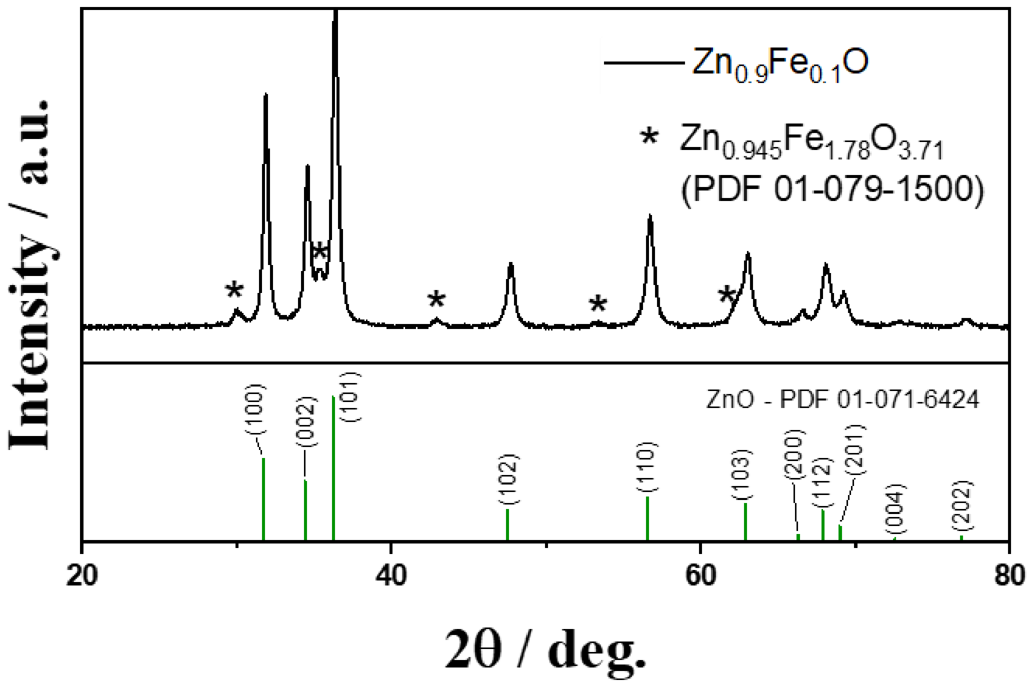

Figure S1. XRD pattern of $Zn_{0.9}Fe_{0.1}O$ nanoparticles obtained by the decomposition of zinc acetate and iron acetate. The PDF reference for hexagonal wurtzite-structured ZnO (PDF 01-071-6424) is provided in the bottom of the figure and phase impurities of spinel zinc iron oxide (PDF 01-079-1500) are marked with an asterisk.

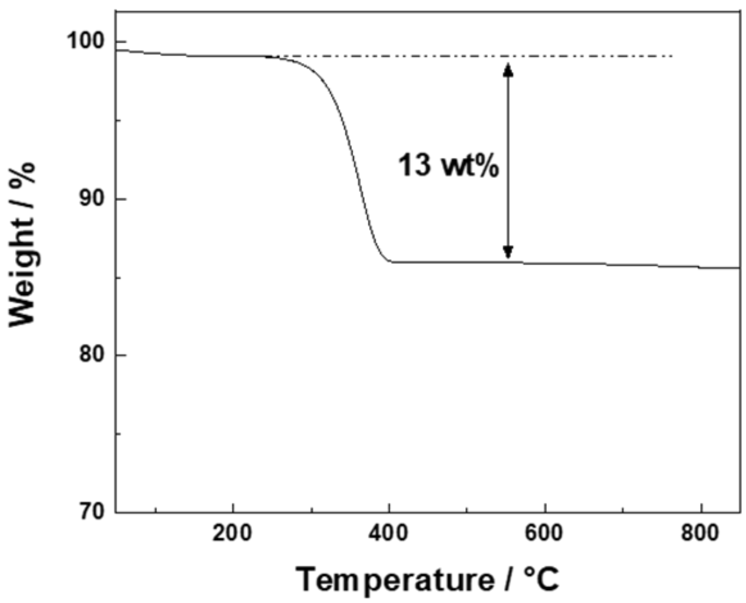

Figure S2. Thermogravimetric analysis of the eventual $Zn_{0.9}Fe_{0.1}O$-C powder under oxygen atmosphere.

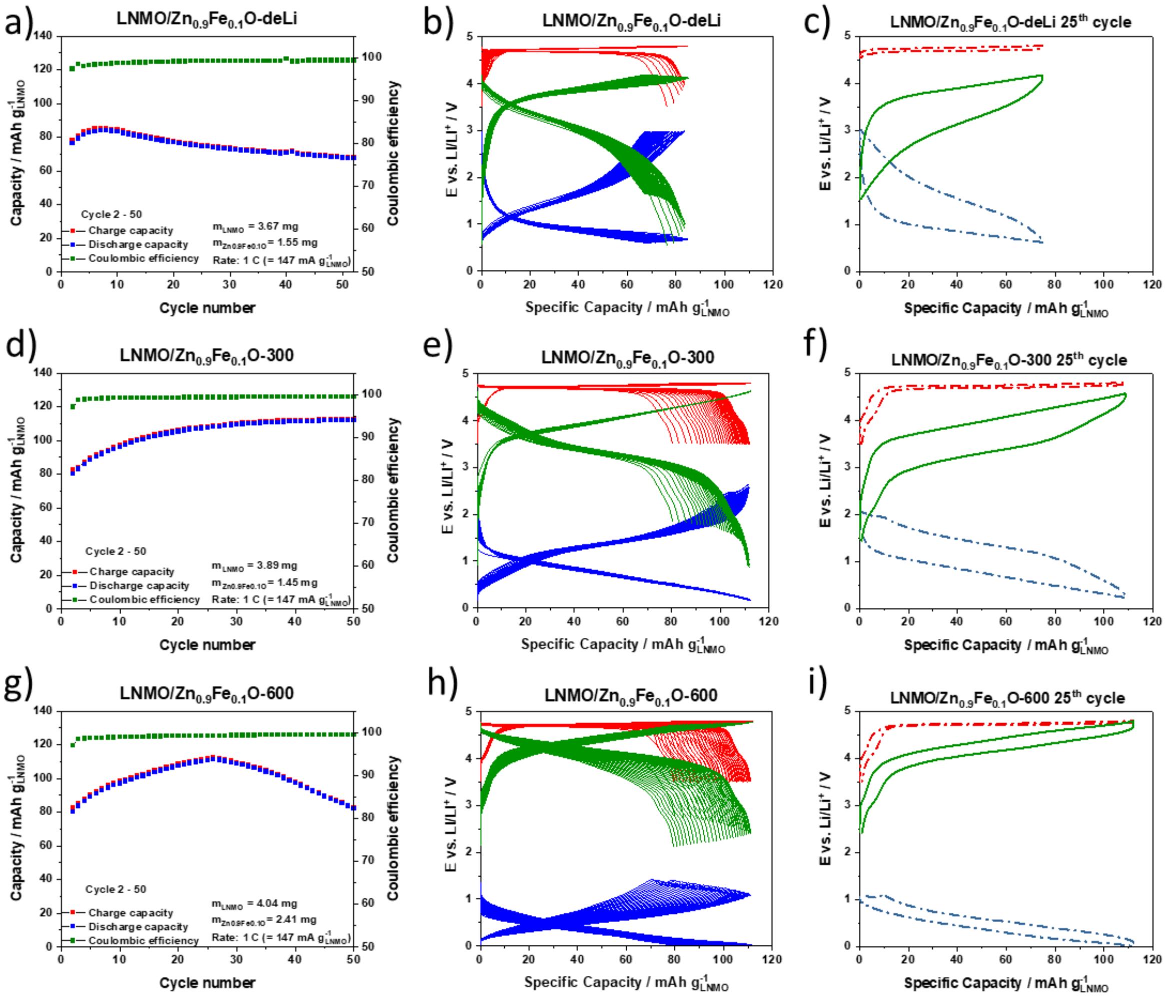

Figure S3. Galvanostatic cycling of $Zn_{0.9}Fe_{0.1}O$/LNMO full-cells at 1C (147 mA g^{-1} (LNMO)) employing anodes with different degrees of pre-lithiation, i.e., (a-c) $Zn_{0.9}Fe_{0.1}O$-deLi, (d-f) $Zn_{0.9}Fe_{0.1}O$-300, and (g-i) $Zn_{0.9}Fe_{0.1}O$-600. For each full-cell the following plots are shown (from the left to the right): the plot of the specific capacity vs. cycle number, the corresponding dis-/charge profiles for all cycles for the full-cell (green), the anode (blue), and the cathode (red) separately, and the slightly modified plot of the dis-/charge profile for the 25th cycle, highlighting the voltage hysteresis between the charge and discharge process. Note that the given specific capacity values are **based on the capacity of the LNMO cathode only**. Prior to cycling at 1C a formation cycle at C/10 was applied to each cell. The potential of the LNMO cathodes and the $Zn_{0.9}Fe_{0.1}O$ anode was limited to 3.5–4.8 V and 0.01–3.0 V, respectively.

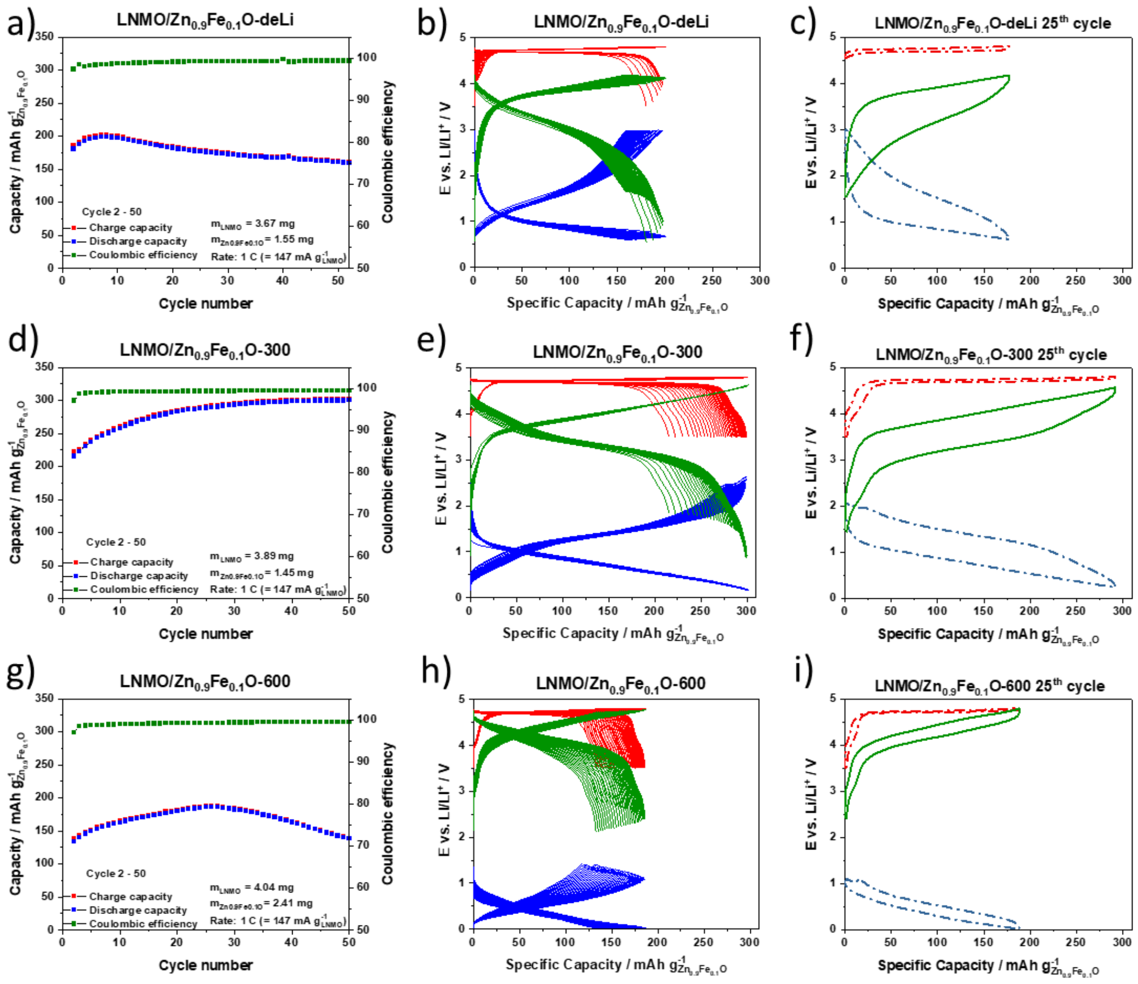

Figure S4. Galvanostatic cycling of $Zn_{0.9}Fe_{0.1}O$/LNMO full-cells at 1C (147 mA g^{-1} (LNMO)) employing anodes with different degrees of pre-lithiation, i.e., (a-c) $Zn_{0.9}Fe_{0.1}O$-deLi, (d-f) $Zn_{0.9}Fe_{0.1}O$-300, and (g-i) $Zn_{0.9}Fe_{0.1}O$-600. For each full-cell the following plots are shown (from the left to the right): the plot of the specific capacity vs. cycle number, the corresponding dis-/charge profiles for all cycles for the full-cell (green), the anode (blue), and the cathode (red) separately, and the slightly modified plot of the dis-/charge profile for the 25th cycle, highlighting the voltage hysteresis between the charge and discharge process. Note that the given specific capacity values are **based on the capacity of the $Zn_{0.9}Fe_{0.1}O$ anode only**. Prior to cycling at 1C a formation cycle at C/10 was applied to each cell. The potential of the LNMO cathodes and the $Zn_{0.9}Fe_{0.1}O$ anode was limited to 3.5–4.8 V and 0.01–3.0 V, respectively.
